# AMPED study: Protocol for a randomized controlled trial of different doses of aerobic exercise training

**DOI:** 10.1097/HC9.0000000000000464

**Published:** 2024-06-19

**Authors:** Jonathan G. Stine, Breianna Hummer, Nataliya Smith, Heather Tressler, J. Westley Heinle, Kyra VanKirk, Sara Harris, Matthew Moeller, Gavin Luzier, Kara DiJoseph, Zeba Hussaini, Ryan Jackson, Brandon Rodgers, Ian Schreibman, Elizabeth Stonesifer, Justin Tondt, Chris Sica, Prashant Nighot, Vernon M. Chinchilli, Rohit Loomba, Christopher Sciamanna, Kathryn H. Schmitz, Scot R. Kimball

**Affiliations:** 1 Department of Medicine, Division of Gastroenterology and Hepatology, Penn State Health—Milton S. Hershey Medical Center, Hershey, Pennsylvania, USA; 2 Division of Gastroenterology & Hepatology, Department of Mediicne, Fatty Liver Program, Penn State Health—Milton S. Hershey Medical Center, Hershey, Pennsylvania, USA; 3 Liver Center, Penn State Health—Milton S. Hershey Medical Center, Hershey, Pennsylvania, USA; 4 Department of Public Health Sciences, The Pennsylvania State University—College of Medicine, Hershey, Pennsylvania, USA; 5 Cancer Institute, Penn State Health—Milton S. Hershey Medical Center, Hershey, Pennsylvania, USA; 6 College of Medicine, The Pennsylvania State University, Hershey, Pennsylvania, USA; 7 Department of Medicine, Penn State Health—Milton S. Hershey Medical Center, Hershey, Pennsylvania, USA; 8 Department of Family Medicine, Penn State Health—Milton S. Hershey Medical Center, Hershey, Pennsylvania, USA; 9 College of Medicine, Center for NMR Research, The Pennsylvania State University, Hershey, Pennsylvania, USA; 10 Division of Gastroenterology and Hepatology, Department of Medicine, University of California San Diego, San Diego, California, USA; 11 NAFLD Research Center, University of California San Diego, San Diego, California, USA; 12 Division of Hematology & Oncology, Department of Medicine, University of Pittsburgh School of Medicine, Pittsburgh, Pennsylvania, USA; 13 Department of Physiology, College of Medicine, The Pennsylvania State University, Hershey, Pennsylvania, USA

## Abstract

Recently renamed, metabolic dysfunction–associated steatotic liver disease remains a leading cause of chronic liver disease worldwide. Regular physical activity is recommended as a treatment for all with this condition because it is highly efficacious, especially when exercise training is undertaken with a specific goal in mind. Despite decades of research demonstrating exercise’s efficacy, key questions remain about the mechanism of benefit and most efficacious dose, as well as the independent impact on liver histology. To answer these questions, we present the design of a 16-week randomized controlled clinical trial of 45 adults aged 18–69 years with metabolic dysfunction–associated steatohepatitis. The primary aim of this study is to better understand the dose required and mechanisms to explain how exercise impacts multiple clinical end points in metabolic dysfunction–associated steatohepatitis. The primary outcome is MRI-measured liver fat. Secondary outcomes include other biomarkers of liver fibroinflammation, liver histology, and mechanistic pathways, as well as cardiometabolic risk and quality of life. This is the first study to compare different doses of exercise training to determine if there is a differential impact on imaging and serum biomarkers as well as liver histology.

## INTRODUCTION

Previously known as NAFLD,^1^ there is no known cure or regulatory agency-approved drug therapy for metabolic dysfunction–associated steatotic liver disease (MASLD), the leading cause of chronic liver disease worldwide.^2^ MASLD develops through multiple factors, including physical inactivity,^3^ and leads to abnormal amounts of fat accumulation in the liver. Low levels of AMP-activated protein kinase (AMPK), an important regulator of hepatic lipogenesis and fatty acid oxidation, promote MASLD development and progression to metabolic dysfunction–associated steatohepatitis (MASH), the more severe variant, with or without liver fibrosis.^4^ If uncorrected, MASH can lead to advanced liver disease or primary liver cancer and may require liver transplantation.^5^

To prevent disease progression, physical activity, including exercise training, which is a subtype of physical activity that is structured, repetitive, and done with a goal in mind,^6^ is recommended for all patients with MASLD.^7,8^ Decades of research have provided substantial evidence supporting the benefit of exercise training in improving biomarkers of liver, cardiometabolic, and oncologic health in individuals with MASLD and MASH.^7–11^ However, despite this large and convincing body of work, how to best prescribe exercise as medicine remains unknown, as reflected by recent clinical practice guidance from the American College of Sports Medicine.^7^ This guidance identified several key knowledge gaps, and highly significant questions remain about exercise’s (1) mechanism of benefit, (2) dose, and (3) impact on liver histology.

The AMPK pathway may hold the key to answering these highly significant questions. AMPK not only plays a large role in global energy balance^12^ but also has a liver-specific role in de novo lipogenesis and fatty acid oxidation. AMPK activity is abnormally low in individuals with MASLD and is partly responsible for the accumulation of excessive amounts of liver fat.^4^ Importantly, exercise is a powerful activator of AMPK. Preclinical models show exercise changes the liver-specific AMPK pathway, leading to less liver fat accumulation mediated by reducing lipogenesis and increasing fatty acid oxidation.^13^ Interestingly, exercise-induced AMPK activation appears dose-related for several reasons: One, as exercise intensity and duration increase and ATP usage increases to the point where it cannot be regenerated quickly enough, AMPK becomes activated.^14^ The greatest AMPK activation is observed with longer durations and greater intensities of exercise.^15^ Two, in order to generate additional ATP during exercise, AMPK is released from glycogen, which is the main energy substrate used during sustained aerobic exercise, leading to AMPK activation (glycogen-bound AMPK is inactive).^16^ AMPK also regulates HSCs and, when stimulated by exercise, may inactivate HSCs and, in effect, prevent liver fibrogenesis.^17^

Prior studies in a small number of individuals with MASH demonstrated that exercise training improved downstream targets of AMPK^18,19^ and that only those individuals with MASLD who performed guideline-based amounts of aerobic exercise, defined as 750 metabolic equivalents of task (MET)-min/wk, achieved clinically meaningful improvement in intermediate biomarkers of liver fibroinflammation.^10^

**FIGURE 1:**
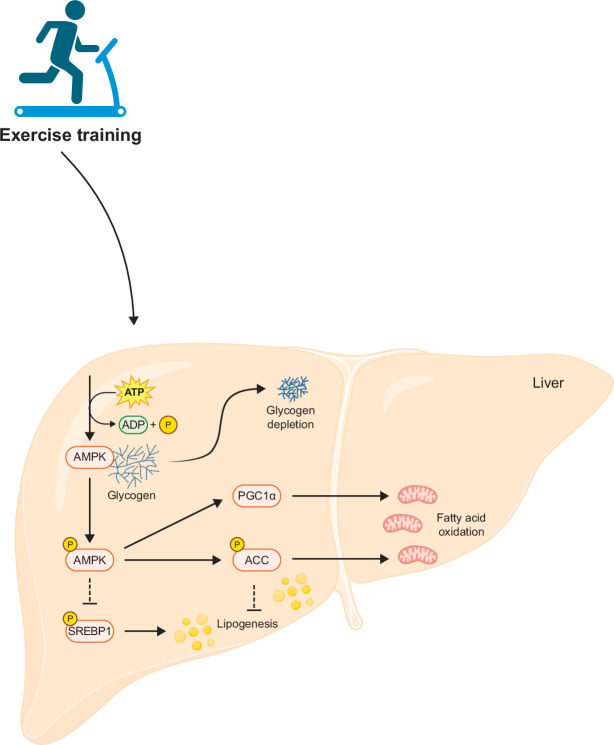
Mechanistic hypothesis of the AMP-activated protein kinase exercise dosing study. Abbreviations: ACC, acetyl-coenzyme A carboxylase; AMPK, AMP-activated protein kinase; PGC1α, peroxisome-proliferator-activated receptor γ coactivator 1α; SREBP1, sterol regulatory element binding protein 1.

Accordingly, through the conduct of the NASH AMP-activated protein kinase Exercise Dosing (AMPED) study, we aim to better understand the dose required and mechanisms to explain how exercise impacts clinical endpoints in MASH. We hypothesize that exercise training will deplete liver glycogen through activation of AMPK and improve biomarkers of liver fibroinflammation and liver histology in adults with MASH (Figure 1). If the anticipated results are achieved, we expect to change clinical practice and revolutionize how exercise training programs are delivered to individuals with MASLD, offering earlier intervention that may arrest, reverse, or even cure this leading cause of chronic liver disease.

## METHODS

### Overview

**FIGURE 2:**
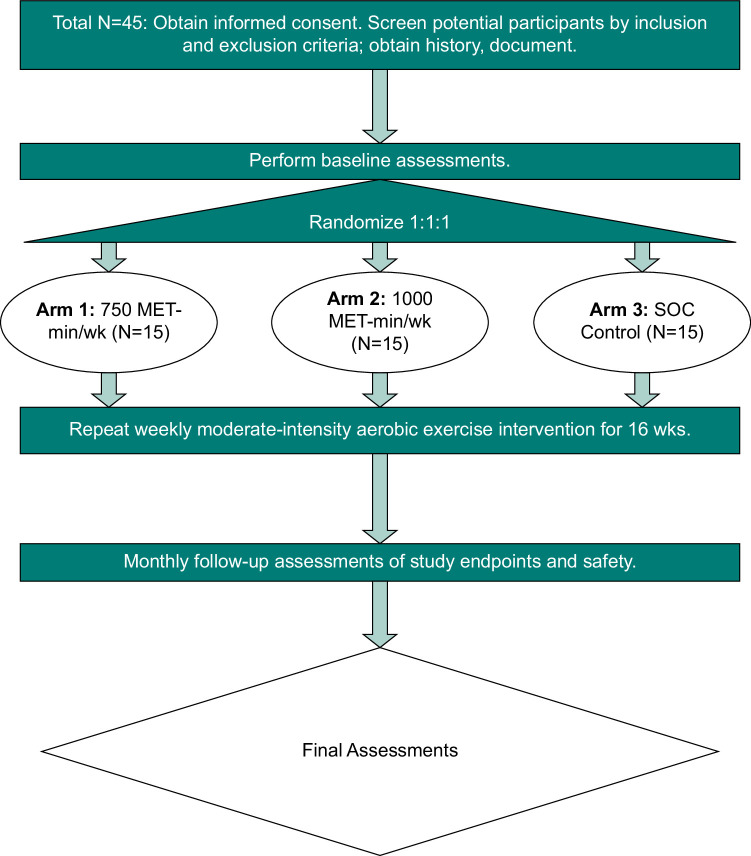
Schema for AMP-activated protein kinase exercise dosing study. Abbreviations: MET, metabolic equivalents of task; SOC, standard clinical care.

The AMPED study is a 16-week single-center, randomized controlled clinical trial (NCT04987879) that seeks to randomize 45 adults between age 18–69 years who have a liver biopsy diagnosing MASH with F1-3 within the preceding 6 months and a body mass index (BMI) of >25 kg/m^2^ to 1 of 3 study arms: arm 1—750 MET-min/wk of aerobic exercise; arm 2—1000 MET-min/week of aerobic exercise or; arm 3—standard clinical care (SOC). An exercise dose >750 MET-min/week was chosen because while it has not been exclusively studied before, it is plausible that higher doses may be even more effective because glycogen depletion requires sustained moderate-vigorous exercise training. Subjects in each group receive dietary counseling every 4 weeks. Protocol compliance is ensured by direct supervision of exercise sessions, either in-person or remotely with audiovisual telehealth, and regular study team interaction. The primary outcome is the MRI proton density fat fraction (PDFF) and based on multiple previous trials,^20–22^ we conservatively expect the 3 study groups (arm 1—750 MET-min/wk vs. arm 2—1000 MET-min/wk vs. arm 3—SOC), to experience mean relative reductions in MRI-PDFF of −30%, −40%, and −5%, respectively. Secondary outcomes include other biomarkers of liver fibroinflammation, liver histology, and mechanistic pathways, as well as cardiometabolic risk. All study procedures are approved by the Institutional Review Board (IRB) at Pennsylvania State University College of Medicine (Study 00018280). Figure 2 summarizes the schema for the AMPED study.

### Eligibility criteria

Table 1 describes the inclusion and exclusion criteria for the AMPED study. This study was designed to select individuals who would derive the greatest benefit from exercise training without experiencing an intervention-related adverse event (AE), such as musculoskeletal injury or major adverse cardiovascular event. Like multiple previous MASLD exercise training trials, we elected to enroll only those individuals who were sedentary (<90 min/wk of self-reported moderate-intensity physical activity). The 6-month window wherein a liver biopsy was required to be obtained prior to randomization was chosen to ensure the most accurate histologic assessment; this interval is a standard inclusion criterion for most major MASH clinical trials.^23^ Only subjects with F1-F3 disease are being enrolled because the change in liver fibrosis was deemed an important secondary end point. To ensure safety of maximal cardiorespiratory fitness testing, subjects are excluded when (1) BMI >45 kg/m; (2) a positive response to the Get Active Questionnaire during prescreening; (3) they are unable to walk ¼ mile or >2 blocks independently; or (4) had an abnormal electrocardiogram (ECG). To avoid confounding from body weight loss, individuals with recent or active weight loss program or supplement use as well as individuals who, within 3 months of study enrollment, have had a dose adjustment of glucagon-like peptide one receptor agonists, including those which are part of a combination therapy (eg, tirzepatide) prescribed for weight loss are excluded.

**TABLE 1:** Inclusion and exclusion criteria for the AMPED study

Inclusion criteria
1) Age 18–69 y at the time of signing informed consent 2) Sedentary (≤90 min/wk of physical activity identified by the GAQ) 3) BMI ≥25 kg/m^2^ 4) Liver biopsy within 6-mo prior to SV showing MASH: a) NASH Clinical Research Network histology scoring system (NAS) ≥4 b) F1-F3 5) MRI-PDFF ≥5%
Exclusion criteria
1) Active cardiac symptoms 2) Active smoking 3) Alcohol use disorder (self-report men >30 g/d or women >20 g/d), AUDIT-C ≥4) 4) BMI >45 kg/m^2^ 5) Cancer that is active 6) Current pregnancy or plans to become pregnant during the study period 7) Exercise deemed unsafe (eg, unable to walk >2 blocks or ¼ mile) 8) History of uncontrolled type 2 diabetes (A1c >9% or changes in diabetes medication doses within 3 mos. of SV) 9) Known or suspected history of drug abuse at the discretion of study investigator 10) Other liver disease (eg, viral hepatitis), including liver transplantation 11) Secondary causes of hepatic steatosis (eg, recent use of steatogenic drugs) 12) Severe medical comorbidities that may hinder study participation at the discretion of study investigator 13) Unable to provide informed consent 14) Vulnerable participants (eg, protected adults under guardianship or committed to an institution by governmental or judicial order)

Abbreviations: AUDIT, alcohol use disorders identification test; BMI, body mass index; GAQ, Get Active Questionnaire; MASH, metabolic dysfunction–associated steatohepatitis; NAS, NAFLD activity score; PDFF, proton density fat fraction; SV, screening visit.

### Recruitment and retention

The AMPED study will recruit 45 adults at a single US-based tertiary care academic medical center with a large multidisciplinary MASLD clinic. Potential subjects are being recruited by study staff from this multidisciplinary clinic as well as from general hepatology clinic. An existing clinical registry of patients with MASLD is an additional resource from which study staff are recruiting potential subjects by phone call. Beyond this, the health system marketing department is assisting with recruitment through multiple approaches including (1) print marketing flyers placed in key locations throughout multiple sites at the health system; (2) recruitment message that plays on the “on-hold” message for the entire academic medical center; (3) daily recruitment messages delivered through multiple social media platforms. A web-based recruitment tool (https://studyfinder.psu.edu) is also being used through academic medical center. We have used these methods successfully before.^11,24^ Potential subjects begin the prescreening process once identified by study staff from the above methods. Study staff review inclusion/exclusion criteria as well as the patient’s electronic medical record to confirm eligibility. If a participant is eligible, they are invited for a screening visit. The participant then enters a screening phase for up to 28 days after eligibility is confirmed with prescreening.

To help with subject retention, compensation is provided at study enrollment, midpoint, and following completion of all end-of-protocol testing for a total of $250. Subjects are also permitted to keep their fitness activity tracker if they elect. Additionally, the option of completing exercise sessions remotely under direct supervision through audiovisual telehealth (eg, Zoom) provides greater flexibility for subjects with time or travel constraints and allows for greater adherence. We have previously validated these methods and found this to be true.^25^ Nonetheless, the AMPED study is powered to accommodate 20% attrition.

### Randomization

Subjects for the AMPED study are randomized 1:1:1 to arm 1 (750 MET-min/wk), arm 2 (1000 MET-min/wk), or arm 3 (SOC) for 16 weeks. To account for potential confounding from diabetes,^26^ stratified randomization has been invoked to ensure equal subjects in each group within the nondiabetes and diabetes stratum. Randomization is automatically performed by the study database (REDCap, Vanderbilt University), a secure, web-based application that supports data capture for research studies and serves as a data repository.^27^

### Measures

Table 2 lists the study measures and their timing. All self-reported measures are completed by participants via tablet computer, smartphone, or paper questionnaire should a digital option not be available for whatever reason.

**TABLE 2:** Schedule of activities

Study phase	Screening (4 wk)	Study intervention period (16 wk)					
Visit	**1**	**2**	**3**	**4**	**5**	**6**	**ED**
Study week	−4 to 0	0	4	8	12	16	—
Visit window (d)	—	+/−1	+/−1	+/−1	+/−1	+/−1	—
Type of visit	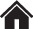	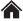	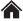	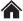	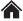	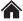	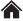
Study procedure							
Informed consent	X	—	—	—	—	—	—
Eligibility criteria	X	X	—	—	—	—	—
Demographics	X	—	—	—	—	—	—
Height	X	—	—	—	—	—	—
Weight and BMI	X	X	X	X	X	X	X
Waist and hip circumference	X	—	X	X	X	X	X
Skinfold measurements^a^	X	—	X	X	X	X	X
Medical history	X	X	X	X	X	X	X
AUDIT-C questionnaire	X	—	X	X	X	X	X
Urine pregnancy test	X	—	—	—	—	—	—
Lifestyle counseling	X	X	X	X	X	X	X
Randomization	—	X	—	—	—	—	—
Fitness tracker set-up	—	X	—	—	—	—	—
Liver biopsy	—	—	—	—	—	X	X
Case report forms	X	X	X	X	X	X	X
Safety							
AEs	X	X	X	X	X	X	X
Physical exam	X	X	X	X	X	X	X
Concomitant medications	X	X	X	X	X	X	X
Vital signs	X	X	X	X	X	X	X
ECG	X	—	—	—	—	X	X
Efficacy							
VCTE	X	—	X	X	X	X	X
MRI-PDFF, MRS	X	X	—	—	—	X	X
Circulating biomarkers^b^	X	—	—	—	—	X	X
AMPK pathway^c^	X	—	—	—	—	X	X
Lipid parameters^d^	X	—	—	—	—	X	X
Body composition^e^	X	—	—	—	—	X	X
Cardiorespiratory fitness	X	—	—	—	—	X	X
Behavioral change							
Dietary intake and counseling^f^	X	—	X	X	X	X	X
Physical activity^g^	X	—	X	X	X	X	X
Adherence	—	—	X	X	X	X	X
MASH counseling	X	—	—	—	—	—	—
Patient-reported outcomes							
PROMIS	X	—	X	X	X	X	X

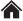
= in-person visit with study staff.

a Skinfolds are obtained at seven sites: chest, abdominal, thigh, subscapular, suprailiac, and midaxillary.

b Liver enzymes (ALT, AST), Adiponectin, CK-18, CRP, ELF, FGF21, FIB-4, IL-6, MASLD comorbidities (A1c, insulin, glucose, HOMA-IR, McAuley Index), PRO-C3,TIMP-1 as well as whole-blood biobanking, NFS, ASCVD score, and Framingham risk.

c AMPK, SREBP1c, ACC, ACADAM, PGC1α, CK-19.

d LDL, HDL, triglycerides.

e Total body fat, visceral adipose tissue, subcutaneous adipose tissue, and fat-free mass as measured by DXA.

f Dietary intake via self-report.

g Fitness tracker measured step counts, self-reported physical activity (IPAQ, DASI).

Abbreviations: AEs, adverse events, AMPK, AMP-activated protein kinase; AUDIT, Alcohol Use Disorders Identification Test; BMI, body mass index; ECG, electrocardiogram; MASH, metabolic dysfunction–associated steatohepatitis; MRS, magnetic resonance spectroscopy; PDFF, proton density fat fraction; PROMIS, Patient-Reported Outcomes Measurement Information System; VCTE, vibration-controlled transient elastography.

### Primary outcome: MRI-PDFF

Participants undergo baseline and postintervention noncontrasted MRI examinations of the abdomen to evaluate alterations in hepatic fat content. Imaging data are acquired using a Siemens 3T PrismaFit system (Siemens Healthineers, Erlangen, Germany) equipped with an 18-channel body flex array and a 32-channel spine coil for signal reception. For the quantification of hepatic fat content, a 2D breath-held multiecho gradient echo sequence is employed to generate PDFF maps. The sequence parameters include a repetition time of 175 ms, echo times ranging from 2.30 to 10.35 ms, echo spacing of 1.15 ms, spatial resolution of 2.08 × 2.08 × 8 mm, and acquisition of 14 slices. Reconstruction of fat, water, in-phase, and opposing phase images is performed automatically at the system console. PDFF maps are reconstructed using Matlab (Mathworks Inc, Natick, MA) with custom software to quantify hepatic steatosis.^28^ Liver segmentation is manually performed using the Insight Segmentation and Registration Toolkit package, separately on both PDFF maps and three-dimensional anatomical images, ensuring the exclusion of major vessels and visceral fat at the liver boundary. PDFF is calculated as the mean PDFF value across the segmented liver.

### Secondary study outcomes

#### Alcohol use

The Alcohol Use Disorders Identification Test-C questionnaire is administered by study staff to assess self-reports of alcohol use.^29^ If, during the study, significant alcohol use becomes apparent, an individualized corrective action plan is to be enacted by study team members to ensure no confounding from unexpected heavy alcohol consumption.

#### Biomarkers of liver fibroinflammation

Serum and imaging biomarkers are measured during the AMPED study. Serum biomarkers include adiponectin, cytokeratin-18, enhanced liver fibrosis, FGF 21, N-terminal type III collagen propeptide, and TIMP-1. Vibration-controlled transient elastography, an imaging-based biomarker, is also being completed.

#### Body composition

Body composition assessment includes dual-energy x-ray absorptiometry (DXA) and skinfold measurements. DXA scans are conducted using the General Electric Healthcare (Chicago, IL) Lunar iDXA DXA scanner, which provides precise evaluation of bone density and comprehensive body composition analysis. The scanner’s CoreScan feature enables accurate quantification of visceral fat. Subjects lie supine during the DXA scan, maintaining stillness and normal breathing, with scan duration ranging from seven to 13 minutes depending on body thickness, automatically adjusted by the scanner based on height and weight input. Skinfold measurements are obtained using a Harpenden Skinfold Caliper (Baty International, England), calibrated to National Standards for reliable metrics (eg, measuring range 0–80 mm, accuracy 99%). Skinfold sites include the chest, abdomen, thigh, triceps, subscapular, suprailiac, and midaxillary regions. Measurements are conducted by a trained study staff member and analyzed using the Harpenden Skinfold Caliper Body Assessment Software for precise assessment. Using a flexible tape measure, hip and waist circumference are also both measured. For waist circumference, the tape measure is placed horizontally around the waist at the narrowest point between the lower rib border and the iliac crest. Hip circumference is measured with a horizontally placed tape measure around the widest part of the gluteus maximus.^30^

#### BMI and weight

Weight (kilogram) is measured with either a Scale-Tronix (White Plains, NY) oversized wheelchair scale or Detecto (Webb City, MO) scale. Height (centimeter) is measured with a portable stadiometer. BMI is calculated by taking weight in kilograms and dividing by the square of height in meters.

#### Cardiorespiratory fitness

All AMPED study participants are required to undergo cardiopulmonary fitness testing following avoidance of caffeine intake or vigorous physical activity in the preceding 24 hours. The subjects underwent a maximal oxygen consumption (VO_2_peak) test utilizing the Trackmaster Treadmill in conjunction with the ParvoMedics’ TrueOne 2400 metabolic measuring system and the Quinton Q-Stress ECG monitor.^31^ The Quinton Q-Stress ECG machine is utilized for monitoring heart rhythms, while the Parvo Medics’ TrueOne 2400 facilitates indirect calorimetry and VO_2_max measurement, incorporating a paramagnetic oxygen analyzer (range: 0%–100%, accuracy: 0.1%, response: 200 ms), an infrared carbon dioxide analyzer (range: 0%–15%, accuracy: 0.1%, response: 100 ms), and a Rudolph heated pneumotach flow/volume measurement (range: 0–800 L/min, accuracy: +/−2% with Precision “Yeh” Algorithm).

Following a 1-min calibration period, which includes a baseline 12-lead ECG, each subject proceeds with the VO_2_peak test following the Bruce treadmill ramp protocol. The safety and feasibility of the Bruce treadmill ramp protocol have previously been established in subjects with MASH.^32^ The protocol consists of sequential stages with incremental speeds and grades. Blood pressure is monitored using a manual blood pressure cuff at the conclusion of each stage until the subject begins running. Subjects are instructed on the utilization of the Borg Rating of Perceived Exertion scale^33^ with measurements taken at the conclusion of each stage. VO_2_peak is determined based on the highest oxygen uptake achieved with maximal heart rate within 10 beats per minute of age-predicted maximum heart rate, and/or a respiratory exchange ratio value exceeding 1.05, and/or an RPE score exceeding 18. Age-predicted VO_2_peak levels are calculated utilizing established formulas for untrained normal weight individuals. VO_2_peak values are expressed in standard units of mLO_2_/kg/min. Upon completion of the VO_2_peak test, a 2–5 min cool-down period is implemented with continuous heart rhythm monitoring and periodic blood pressure assessments. The treadmill is stopped once the subject’s heart rate has sufficiently returned to baseline levels. Discontinuation of all maximal tests is determined by the onset of symptoms, and the presence of a physician and a study exercise physiologist is ensured throughout all VO_2_peak tests.

#### Dietary assessment

Dietary composition is measured at baseline for all subjects. At the screening visit, subjects self-report their dietary intake via a 24-hour dietary recall form as well as a food frequency questionnaire. Once randomized, subjects self-report their daily dietary intake securely via the FitBit smartphone application, which automatically calculates caloric, macronutrient, fiber, and sodium intake. On a monthly basis, study staff reviews the self-reported dietary intake and confirms the automatically calculated nutrition information. To confirm the accuracy of self-reports, participants are asked to submit a photograph of a random meal at different timepoints throughout the AMPED Trial. Study team members cross-reference the photographic meal evidence with self-reported intake to confirm accuracy. A total of 16 photographs (4 breakfast, 4 lunch, 4 snack, and 4 dinner) are being submitted during the 16-week study period.

#### Laboratories

The following laboratories are being obtained during the AMPED study following an overnight fast of at least 8 hours in length: albumin, alanine aminotransferase, alkaline phosphatase, aspartate aminotransferase, bilirubin (total), cholesterol (total), c-reactive protein, glucose, hemoglobin A1c, HDL, LDL, insulin, IL-6, total protein, and triglyceride. Homeostatic Model Assessment of Insulin Resistance^34^ is calculated to evaluate insulin resistance as (Fasting insulin)×(Fasting glucose)/405. The McAuley Index will also be calculated to measure insulin resistance as exp[2.63 − 0.28×ln(Insulin (mU/L) − 0.31×ln(triglyceride (mmol/L)].^35^ From the appropriate laboratories, MASH clinical decision aids NAFLD Fibrosis Score and Fibrosis-4 index are being calculated.^36^ To determine cardiovascular risk, the Atherosclerotic Cardiovascular Disease score and Framingham risk score are also calculated. Whole blood is being biobanked as well.

#### Liver histology

An independent blinded liver pathologist is determining the NAFLD Activity Score^37^ and liver fibrosis stage. This is then compared to the clinical biopsy report. Interobserver agreement is determined by a formal Kappa calculation (>0.7 will be considered agreement). The AMPK pathway will be analyzed from the liver biopsy specimen. From liver tissue, the abundance of specific mRNAs will be assessed by Predesigned TaqMan Real-time PCR assays using our current methods.^38^ Specimens will be placed in RNA*later* Stabilization Solution. RNA will be isolated using a PureLink RNA Mini Kit. RNA quality will be assessed (Agilent 2100 Bioanalyzer) as will the abundance of specific mRNAs (eg, Acetyl-CoA carboxylase 1, sterol regulatory element-binding protein-1, carbohydrate-responsive element-binding protein, peroxisome proliferator-activated receptor-γ coactivator 1α, acyl-CoA dehydrogenase medium chain). RNA will be stored as ethanol precipitates (−20 °C) and protein (−80 °C) after boiling in SDS sample buffer until analysis. AMPK activation and AMPK (both phosphorylated and unphosphorylated) as well as Acetyl-CoA carboxylase 1 protein expression will be assessed by western blot.^39^ Approximately 50 mg of tissue will be homogenized in buffer containing a mixture of protease and RNase inhibitors. After centrifugation, equal amounts of protein will be resolved on SDS polyacrylamide gels and transferred to PVDF membrane. The membrane will be stained to confirm equal protein loading, destained, and then blocked (5% nonfat dry milk). The membrane will be probed with validated primary antibodies (followed by secondary antibody and visualized using Clarity Western ELC Blotting Substrate. Protein abundance will be expressed relative to either total protein or, for phospho-proteins, to the total amount of the specific protein. AMPK activation will be assessed by western blot using anti-phospho-AMPK substrate motif antibody.

We will assess colocalization of glycogen and AMPK by immunofluorescence microscopy using a fusion protein (GYSC) containing glutathione S-transferase, a cMyc epitope and the carbohydrate-binding module from starch-binding domain-containing protein 1, for use as a glycogen-binding probe.^40^ The GYSC probe has a glutathione s-transferase-tag that will be visualized using a fluorescent anti-glutathione s-transferase antibody. A fluorescently tagged anti-rabbit antibody will be used to visualize AMPK. The results of this analysis are expected to provide direct support for the hypothesis that exercise-induced depletion of liver glycogen leads to dissociation of AMPK from glycogen and its subsequent activation. While we expect to be able to localize glycogen signal to the hepatocytes based on cell morphology, we will also stain with a fluorescently labeled antibody against hepatocyte-specific antigen as confirmation. Because AMPK activation can inactivate fibrogenic HSCs, we will perform additional exploratory analysis using mRNA extraction and real-time PCR to measure αsmooth muscle actin, peroxisome proliferator-activated receptorγ, and collagen-1α, which are validated markers of HSC activity.^41^

#### MR-glycoCEST

In order to standardize glycogen measurement via glycoCEST,^42^ we are using the following validated methods as a part of the study protocol.^43^ On the day prior to metabolic and glycogen testing, all subjects are instructed to consume 55% of their calories from carbohydrates. On the evening prior, subjects are provided a standard final meal consumed the evening before testing to standardize macronutrient intake across participants (Nestle Boost Very High Calorie, 237 mL, Nestle Inc, Bridgewater, NJ). Subjects submit a photo to research staff to document their completion of the shake. Subjects then perform their exercise session the next morning followed immediately by completion of glycoCEST measured glycogen of both the liver and the calf muscle. Given the interplay between the liver and the skeletal muscle, it is important to examine this relationship in greater detail as depletion of muscle glycogen may lead to further mobilization of liver glycogen as a source of glucose that can be delivered via the bloodstream for exercise performance. For this reason, we have decided to measure calf skeletal muscle glycogen with glycoCEST (at the same timepoints that liver glycoCEST glycogen measurement is occurring) as this is a validated, reliable noninvasive method.^42^ The time between final meal and exercise session is standardized and is no longer than 12 hours, given this is the amount of time it takes for fasting to deplete liver glycogen.^44^

GlycoCEST measurements is being performed using the same Siemens 3T PrismaFit system (Siemens Healthineers, Erlangen, Germany) that will be used to measure MRI-PDFF. A single-slice chemical exchange saturation transfer (CEST) sequence is employed to quantify the glycoCEST effect. Sequence parameters in the liver include a repetition time of 4200 ms, spatial resolution of 2.5×2.5×8 mm, CEST preparation duration of 500 ms, CEST preparation amplitude of 1.25 uT,^45^ and the z-spectrum are sampled from −4 to 4 PPM with 32 samples, including a S0 image. In the calf muscle sequence parameters include a repetition time of 7000 ms, spatial resolution of 1.25×1.25×8 mm, CEST preparation duration of 500 ms, CEST preparation amplitude of 1 uT,^42^ and the z-spectrum are sampled from −4 to 4 PPM with 41 samples, including a S0 image.

The CEST z-spectrum data are analyzed offline with custom software developed in Matlab, from which the mean MTR asymmetry value will be calculated in select region of interests in the liver tissue and calf skeletal muscle. A previously described technique^45^ is being utilized for the correction of the liver z-spectrum data. The simultaneous mapping of the water shift and B1 technique^46^ is being used to correct the calf muscle z-spectrum data.

#### Physical activity and functional capacity assessment

To assess all physical activity and not just that completed as part of the exercise training program, the International Physical Activity Questionnaire^47^ is being administered. International Physical Activity Questionnaire questions pertain to different physical activity domains including home, job-related, sitting, sport, and transportation. Additionally, fitness activity trackers are being provided to each subject to allow for remote monitoring 24 hours a day, 7 days a week. The fitness tracker (FitBit Charge HR, FitBit, San Francisco, CA) has a heart rate monitor and can track step counts, flights of stairs climbed, total distance walked, total duration of activity based on custom heart rate zones established after cardiorespiratory fitness testing, calories expended from physical activity, and resting heart rate. Fitness trackers are also being used to monitor remote exercise sessions if a subject elects to perform this type of session, but only after the approval of the study staff. Physical activity information is being downloaded and reviewed monthly by study staff members. To address functional capacity, the Duke Activity Status Index^48^ questionnaire is also being administered to augment physical activity capture and better understand how physical activity and fitness translate to everyday life for each subject.

#### Quality of life

Because individuals with MASLD exhibit diminished quality of life across all health domains,^49^ subjects in the AMPED study are undergoing assessment using the Patient-Reported Outcomes Measurement Information System Computerized Adaptive Testing tool via tablet computer (iPad). Participants are prompted to reflect on their experiences over the past week in nine distinct health domains, including anxiety/fear, cognitive function, depression/sadness, fatigue, instrumental support, pain interference, physical function, sleep disturbance, and social roles. A standardized protocol is implemented to elucidate the assessment process to participants and offer direct assistance with any technical challenges pertaining to the tablet computer. Our previous work has demonstrated the validity and security of utilizing tablet computers by individuals with MASH performing an exercise intervention.^11^ Real-time scores for each health domain are automatically computed and normalized to the underlying population distribution derived from responses obtained during the 2000 US Census utilizing T-score algorithms provided by the Patient-Reported Outcomes Measurement Information System Assessment Center software.^50^ The median T-score is established at 50, with an SD equivalent to 10.

#### Vital signs

Blood pressure (mm Hg) and heart rate (bpm) are being measured once the subject sits at rest for at least 5 minutes. Blood pressure is obtained using an automated, portable blood pressure and heart rate monitor (Omron HEM 907XL)^51^ or a manual sphygmomanometer (Welch Allyn, Skaneatles Falls, NY). Resting heart rate is obtained by a fitness activity tracker with a heart rate monitor (FitBit, San Francisco, CA). Temperature is measured using a Genius Tympanic Ear Thermometer with Base LCD Display (Cardinal Health, Dublin, OH).

### Covariates

AMPED study participants directly report, or study staff verify self-reported information in the electronic health record documenting their demographics (eg, age, gender, race/ethnicity, education). Past medical, surgical, and social history are reviewed, including smoking status and history of illicit drug use. Medications are also reviewed both from self-report and the electronic health record, including those that are prescribed to treat MASH. Table 3 summarizes captured outcomes and covariates.

**TABLE 3:** Study objectives and end points

End point	Methods
Primary efficacy	
Liver fat	• MRI-PDFF will assess change in liver fat after week 16
Secondary efficacy—key	
Liver and muscle glycogen	• MR-S will assess change in liver and muscle glycogen after week 16
Liver fat and liver stiffness using imaging analysis	• VCTE will assess change in liver fat and stiffness after week 16
Clinically meaningful improvement in liver fat and stiffness	• Proportion of participants with ≥26% relative reduction in VCTE-measured liver stiffness (kPA) after week 16 • Proportion of participants with ≥30% relative reduction in MRI-PDFF after week 16
Circulating biomarkers of hepatic injury	• Change in ALT, AST, CK-18, CRP, IL-6 after week 16 • Proportion of participants with ≥17 IU/L decrease in ALT after week 16
Circulating biomarkers of liver fibrosis and fibrogenesis	• Change in ELF score, Fibrosis-4 index (FIB-4), NFS, PRO-C3, TIMP-1 after week 16
Secondary efficacy—other	
Lipid parameters	• Change in LDL, HDL, triglycerides after week 16
Biomarkers for MASLD comorbidities	• Change in Hemoglobin A1c, insulin, plasma glucose, HOMA-IR, McAuley Index, ASCVD, Framingham score after week 16
Body composition	• Change in total body fat, visceral adipose tissue, subcutaneous adipose tissue, and fat-free mass as measured by DXA after week 16 • Change in waist and hip circumference, skinfolds at weeks 4, 8, 12, 16
Dietary intake and physical activity	• Change in fitness tracker measured step counts, self-reported physical activity (IPAQ, DASI), self-reported dietary intake at weeks. 4, 8, 12, 16
Patient-reported outcomes	• Change in PROMIS after week 16
Adherence	• % of completed exercise sessions after week 16 • % who achieved a priori definition of adherence (>80% session completion) after week 16
Safety	
Safety and tolerability	• Safety and tolerability will be evaluated in terms of adverse events (AE), physical exam, and changes in body weight and/or vital signs at weeks 4, 8, 12, 16

Abbreviations: AE, adverse event; ALT, alanine aminotransferase; Apo, apolipoprotein; ASCVD, atherosclerotic cardiovascular disease; AST, aspartate aminotransferase; CK, cytokeratin; CRP, C-reactive protein; DASI, duke activity score index; DXA, dual x-ray absorptiometry; ELF, enhanced liver fibrosis; FIB-4, Fibrosis-4 index; HOMA-IR, homeostatic model assessment for insulin resistance; IPAQ, international physical activity questionnaire; MASLD, metabolic dysfunction–associated steatotic liver disease; MASH, metabolic dysfunction–associated steatohepatitis; MR-S, magnetic resonance spectroscopy; NFS, NAFLD Fibrosis Score; PDFF, proton density fat fraction; PRO-C3, N-terminal type III collagen propeptide; PROMIS, Patient-Reported Outcomes Measurement Information System; VCTE, vibration-controlled transient elastography.

## STUDY INTERVENTIONS

The AMPED study is a 16-week randomized, controlled interventional trial designed to test the hypothesis that exercise training will deplete liver glycogen through activation of AMPK and improve biomarkers of liver fibroinflammation and liver histology in adults with MASH.

### Exercise training (Arms 1 and 2)

American College of Sports Medicine (ACSM) Guidelines for Exercise Prescription,^52^ including the frequency, intensity, time and type principles, are being used to teach subjects how to perform moderate-intensity aerobic exercise training (40%–59% maximal heart rate). To ensure physiologic adaptation, we included a progressive lead-in period until the target exercise dose is reached by week 4 (Table 4). This successfully prevented injury in our previous exercise intervention in sedentary individuals with MASH.^11^ Aerobic exercise can be completed by walking, jogging, or with cardio equipment (eg, recumbent bike). To further prevent injury, each session includes a warm-up and cool-down with low intensity walking and dynamic stretches. The warm-up is 10 minutes in length and is composed of 5 minutes of walking at 30%–40% of maximal heart rate followed by 5 dynamic exercises including knee to chest, 10-yard lateral shuffle, bent over twist, calf sweeps, and leg swings. Each exercise session ends with a 5-minute cool down on the treadmill at 30%–40% of maximal heart rate.

**TABLE 4:** Acclimation period of progressive exercise for W1–W4

Study arm (MET-min/wk)	Week 1	Week 2	Week 3	Week 4
Arm 1 (750)	300	450	600	750
Arm 2 (1000)	300	500	750	1000

Abbreviation: MET, metabolic equivalents of task.

The study exercise physiologist is revising the program as fitness changes; subjects progress in speed or difficulty to ensure correct exercise intensity. Every exercise session is directly supervised (either in-person or with A-V telehealth) by research staff with real-time heart rate monitoring. After each session, the completed exercise dose is calculated by intensity (METs) × frequency (1 session) × time (min). For example, a typical 45-minute session composed of (10-minute warm-up × 3.0 METS) + (30-minute brisk walking × 5.0 METs) + (5-minute cool down × 3.0 METs) =200 METs. From there, the study exercise physiologist can vary the frequency, intensity, time and type prescription to design the next session (Table 5). For example, if a subject only has 30 minutes, exercise intensity may be increased to slow jogging (6.0 METs) to progress toward the assigned dose. This format is repeated until the assigned summative weekly dose is achieved. At the conclusion of the 16 weeks intervention period, subjects develop a plan to continue to exercise after the trial ends with the assistance of the staff exercise physiologist.

**TABLE 5:** FITT prescription for each exercise study arm

Study arm (MET-min/wk)	Frequency (d)	Intensity	Time (min)	Type
Arm 1 (750)	3–5	Moderate-vigorous	22–45	Aerobic
Arm 2 (1000)	3–5	Moderate-vigorous	30–60	Aerobic

Abbreviation: MET, metabolic equivalents of task.

### SOC (arm 3)

This group is receiving the best MASH clinical practices in accordance with practice guidelines.^53^ Because in-office hepatology counseling occurs every 12–24 weeks,^54^ standard counseling is happening at baseline and end-of-trial and is reinforced by American Liver Foundation handouts. This counseling includes information about completing at least 150 min/wk of physical activity as recommended by the ACSM.^8^ Physical activity is being measured remotely by fitness activity trackers like that in arms 1 and 2. Subjects randomized to SOC are given 16 weeks of fitness center membership and access to the study exercise physiologist for training sessions following completion of the study protocol.

### Dietary counseling

All subjects (arms 1–3) receive dietary counseling every 4 weeks per best clinical practices.^55^ A study Registered Dietitian will review food logs and provide energy expenditure goal-informed feedback. Exclusion of MASLD-promoting components (eg, beverages high in added fructose) is being reinforced. Macronutrient composition is being adjusted according to the Mediterranean diet.

### Adherence to exercise intervention

Adherence is meticulously monitored through various means. Each exercise session is directly supervised, with real-time heart rate monitoring facilitated via the fitness activity tracker. Following each session, study personnel meticulously complete a tracking sheet, which is thoroughly reviewed and subsequently entered in the database. This includes both the minutes completed and the METs. The objective of this monitoring protocol is to attain a total exercise dose each week at the assigned amount. Additional exercise beyond the prescribed in-person sessions is prohibited by the study protocol. Compliance with this stipulation is monitored through remote activity tracking facilitated by fitness tracker devices, with data downloads enabling surveillance. In cases where a subject is suspected of engaging in additional exercise based on this data analysis, they undergo an interview with study personnel for verification. Subsequent re-education is provided as deemed necessary to uphold adherence to the study protocol.

### Sample size and power calculations

We conservatively expect our 3 groups (SOC control vs. 750 MET-mins/wk vs. 1000 MET-mins/wk), to experience mean relative reductions in MRI-PDFF of −5%, −30%, and −40%, respectively, with a SD of 6% (30% or greater is considered clinically significant because this surrogates for histologic improvement).^56,57^ We based these assumptions on (1) our recent meta-analysis of 551 individuals with MASLD from 14 exercise-intervention studies for which −36% and −46% MRI-measured liver fat reduction was observed for 750 MET-min/wk, and 1000 MET-min/wk of exercise, respectively, alongside a +7% gain for SOC controls;^10^ and (2) our NASHFit Trial that found subjects who performed 750 MET-min/wk to experience a −30% reduction in MRI-PDFF compared to +11% for SOC control subjects.^11^ Lower doses of exercise were not chosen because <750 min/wk was found to have similar efficacy as SOC. Additionally, based on our previous experience, we also account for 20% of the subject drop-out in the power calculation, although we fully expect it to be much less.^58^ Based on pairwise comparisons with two-sided *t* tests in an ANCOVA setting, each with a significance level of 0.017 (Bonferroni correction), a sample size of 15 per group (total sample size=45) yields at least 90% statistical power for each pairwise comparison.

### AEs and data and safety monitoring plan

All AEs are systematically recorded by study staff on an ongoing basis and undergo a thorough review by the Principal Investigator to ascertain any potential association with the intervention. Following each exercise session, AEs are actively screened by study staff, which assesses for the occurrence of any AEs or identify barriers hindering the subject’s continuation in the exercise protocol. Furthermore, these screenings are repeated during monthly check-ins with a study investigator. Oversight of this process is provided by an independent medical monitor, who is tasked with ensuring the safety of the study protocol. The PI and the medical monitor convene bi-annually for comprehensive discussions. Prior to each meeting, the medical monitor is furnished with an AE log for review. Within 1 week following the meeting, the medical monitor provides the investigators with conclusions drawn from the discussions. In the event of serious AEs surpassing an anticipated threshold, as determined by the independent medical monitor, further enrollment in the study is halted until an independent local regulatory board, comprising a Hepatologist, a Gastroenterologist, and another uninvolved physician, reaches a unanimous decision to continue the study with appropriate modifications, which are then subject to approval by the local IRB.

For the assurance of data quality, a trained member of the study team conducts bi-weekly data quality checks within the REDCap platform. These checks are subsequently reviewed on a quarterly basis by an independent data quality monitor within the Department of Public Health Sciences. Both the designated study team member and the independent data quality monitor collaborate in advising the research team on matters pertaining to participant accrual, retention, and the completeness of the data collected.

### Data analysis plan

We will invoke the intent-to-treat principle for the primary analyses. We will apply an ANCOVA to compare the 3 groups with respect to the MRI-PDFF (primary outcome) and all other continuous secondary outcomes while including the baseline measurement and diabetes status as covariates. We impose a Bonferroni-corrected significance level of 0.017 for the multiple pairwise comparisons of the three groups. We will apply a Mantel-Haenszel test to compare the 3 groups with respect to dichotomous outcomes while accounting for the baseline measurement and diabetes status. A *p*-value of <0.05 will be regarded as statistically significant. As a series of secondary analyses and sensitivity analyses, we will use multiple imputations for estimating missing values in the outcomes variables to assess the robustness of the primary analyses. We will conduct all statistical analyses via SAS version 9.4 (Cary, NC).

### Planned subgroup analysis

Several subgroup analyses are planned. First, a comparison between diabetics and non-diabetics is planned as diabetes is one of the strongest predictors of more advanced MASH and also treatment response with lifestyle changes.^59^ Second, comparisons across sex/gender and racial/ethnic groups.

## POSSIBLE LIMITATIONS

There are several potential limitations to the design of the AMPED study. One, it is unknown if 1000 MET-min/wk is feasible. However, based on multiple smaller studies,^60,61^ we believe higher doses and intensities of exercise are feasible because sedentary patients with MASH experienced no AEs and low drop-out (~10%), even when >1000 MET-min/wk of vigorous exercise was completed. To further ensure feasibility, we use similar methods, including exercise progression, to prevent injury. We improve upon these methods by using direct supervision and exercise individualization. If adherence issues arise, we will administer our “Barriers to Exercise” tool^62^ to develop an individualized corrective action plan. It is also plausible that even if 1000 MET-min/wk of exercise is feasible, there may be a threshold effect, and in fact, no dose-related relationship may be found. Two, paired liver biopsies may be a potential barrier to enrollment; however, all pilot subjects who were approached to complete a liver biopsy feasibility study after exercise agreed no AEs were observed. Because there are expected intermediate histologic benefits after 16 weeks of exercise, we feel the risks of biopsy are justified.^63^ Three, we do expect our intervention to be weight-neutral as it is similar to our recently published NASHFit study^11^; however, it may be possible for a subject to decrease their carbohydrate intake as the dietary counseling will be in accordance with the Mediterranean diet, including the avoidance of processed food or beverages high in added fructose. In the NASHFit study,^11^ we did not find any significant changes in absolute or relative carbohydrate intake. Based on this, we do not anticipate any significant changes in carbohydrate intake or weight loss, however, if this does unexpectedly occur, we will account for this with statistical modeling and the planned subgroup analysis as above.

## CONCLUSIONS

The AMPED study is being conducted to decipher the required therapeutic dose of exercise and the underlying mechanisms explaining how exercise training leads to changes in biomarkers of fibroinflammation and histologic end points in individuals with MASH. Following the completion of this trial and dissemination of the anticipated findings, multiple highly significant changes in the health of individuals with the leading cause of chronic liver disease worldwide can be expected. One, clinicians will be able to effectively direct and provide exercise resources by prescribing a specific dose of exercise. This is important because only 10%–20% of patients with MASLD achieve the recommended amounts of exercise despite almost all having a desire to be more active.^62^ Inactivity is commonly attributed to insufficient direction about how to exercise or resource provision from treating clinicians.^62^ Two, exercise professionals will have disease-specific guidance to inform individualized exercise prescriptions. By establishing the dose of exercise needed, exercise prescription can be tailored by varying frequency, intensity, time, or type to achieve this needed dose.^52^ A precision exercise prescription to fit each person’s lifestyle while meeting weekly exercise goals offers promise to improve adherence. Doing so will remove other common barriers we previously identified, including lack of time to complete long sessions or physical discomfort with certain exercise types.^62^ Three, novel therapeutic targets may be uncovered by studying how the liver-specific AMPK pathway adapts to exercise at various parts of the pathway. Because exercise can modify multiple hepatic pathways, banked samples from this trial can be studied in the future to measure changes in alternative pathways of interest and using unbiased RNA sequencing to generate additional hypotheses by identifying novel candidate genes involved in exercise response. Lastly, the AMPED study can inform future trial design by beginning to investigate the impact of exercise training on histologic outcomes. Directly measuring exercise’s impact on liver histology is important because histologic response is the best determinant of clinical outcomes and the goal of all late-phase MASH clinical trials.^64^
